# Effectiveness of the school‐based internet intervention *StresSOS* for the prevention of mental health problems in young people: a randomized controlled trial as part of the ProHEAD consortium

**DOI:** 10.1111/jcpp.70145

**Published:** 2026-03-13

**Authors:** Laya Lehner, Vera Gillé, Markus Moessner, Sabrina Baldofski, Stephanie Bauer, Katja Becker, Silke Diestelkamp, Alisa Hiery, Michael Kaess, Julian Koenig, Sophia Lustig, Christine Rummel‐Kluge, Rainer Thomasius, Heike Eschenbeck, the ProHEAD Consortium

**Affiliations:** ^1^ Department of Educational Psychology and Health Psychology University of Education Schwäbisch Gmünd Schwäbisch Gmünd Germany; ^2^ Center for Psychotherapy Research University Hospital Heidelberg Heidelberg Germany; ^3^ Department of Psychiatry and Psychotherapy, Medical Faculty Leipzig University Leipzig Germany; ^4^ Institute of Psychology Heidelberg University Heidelberg Germany; ^5^ German Center for Mental Health (DZPG), Partner Site Mannheim/Heidelberg/Ulm Mannheim/Heidelberg/Ulm Germany; ^6^ Department of Child and Adolescent Psychiatry, Psychosomatics and Psychotherapy University Hospital of Marburg and Philipps‐University Marburg Marburg Germany; ^7^ Center for Mind, Brain and Behavior (CMBB), Philipps‐University Marburg and Justus Liebig University Giessen Marburg Germany; ^8^ University Hospital Hamburg‐Eppendorf German Center for Addiction Research in Childhood and Adolescence Hamburg Germany; ^9^ Department of Child and Adolescent Psychiatry, Centre for Psychosocial Medicine, University Hospital Heidelberg Heidelberg Germany; ^10^ University Hospital of Child and Adolescent Psychiatry and Psychotherapy, University of Bern Bern Switzerland; ^11^ Department of Child and Adolescent Psychiatry, Psychosomatics and Psychotherapy Faculty of Medicine and University Hospital Cologne, University of Cologne Cologne Germany; ^12^ Department of Psychiatry and Psychotherapy, University of Leipzig Medical Center Leipzig Germany

**Keywords:** Online, internet, prevention, intervention, adolescents, young people, mental health, mental illness

## Abstract

**Background:**

Given the high prevalence of mental illnesses in adolescents, there is an urgent need for effective prevention strategies. The aim of this study was to evaluate the school‐based internet intervention *StresSOS* for the universal prevention of mental illnesses in youth.

**Methods:**

A two‐arm, randomized controlled trial was conducted. Participants were recruited from schools across five regions of Germany. Young people between the ages of 12 and 25 years without mental health problems were invited to the trial and randomly assigned to *StresSOS* or to the attention placebo control condition, stratified by sex. Participants in both conditions received eight web‐based sessions with information and exercises and weekly e‐mail teasers about program content and a monitoring survey. *StresSOS* comprised content on life skills, particularly stress management and mental health literacy, and the control condition content comprised healthy nutrition. The primary outcome was self‐reported mental health status at a 12‐month follow‐up. Intention‐to‐treat analyses were calculated. The trial was preregistered with the German Register of Clinical Trials (DRKS00014693, see https://drks.de/search/en/trial/DRKS00014693).

**Results:**

A total of 5,268 eligible students were invited to participate, 2,327 (44%) activated their account and were randomized to *StresSOS* (*n* = 1,154) or to the control condition (*n* = 1,173). Due to COVID‐19‐related school closures, 1,209 were lost to follow‐up, and data from 1,118 students were analyzed (535 in *StresSOS* and 583 in the control group). Participation in *StresSOS* led to significantly reduced incidences of emerging mental health problems at the 12‐month follow‐up (controls: *n* = 162 [28%] ‘with problems’; *StresSOS*: *n* = 113 [21%] ‘with problems’; OR 0.70, 95% CI [0.52, 0.92], *p* = .01).

**Conclusions:**

*StresSOS* was effective in universally preventing the onset of mental health problems, with a small effect. Internet interventions have the potential to contribute to a reduction of the disease burden in young people.

## Introduction

Good mental health is crucial for young peoples' well‐being and their subsequent development (WHO, 2021a). However, every sixth young person suffers from mental health problems (Klipker, Baumgarten, Göbel, Lampert, & Hölling, 2018; Polanczyk, Salum, Sugaya, Caye, & Rohde, 2015). Most mental disorders begin in adolescence, with the highest incidence rates occurring between the ages of 14–25 years (Solmi et al., 2022). In terms of lifetime prevalence, 34.5% of all mental disorders are manifested by the age of 14 years, and 62.5% by the age of 25 years (Solmi et al., 2022). Mental health problems in youth can have a negative impact on educational achievement (Evensen, 2019), physical health (Schlack, Peerenboom, Neuperdt, Junker, & Beyer, 2021), and quality of life (Schlack et al., 2021). Furthermore, they are associated with an increased risk of mental illness in later life, and with high healthcare costs (Whiteford et al., 2013). Thus, the need for effective and broadly available prevention and health promotion programs is evident.

Reviews have shown that online interventions have the potential to prevent mental illnesses and, thus, to contribute to a reduction in overall disease burden (Clarke, Kuosmanen, & Barry, 2015; Noh & Kim, 2023). They may be superior to traditional face‐to‐face programs (for the latter, see, e.g., van Loon et al., 2020) in terms of saving personnel or time resources (e.g., teaching time in schools). With their low‐threshold access, they can be used at any time and from anywhere. This accessibility implies that such interventions may offer a scalable, low‐cost approach to preventing mental disorders. However, as a disadvantage, only around one third of adolescents used an online prevention program at home in their free time (Fridrici & Lohaus, 2009).

Schools provide an institutional setting in which prevention programs can be meaningfully embedded (WHO, 2021b). An entire cohort can be reached and for research, schools offer an organizational structure that enables informed parental consent to be obtained in a reliable manner. In the present study, a school‐based internet intervention *StresSOS* was developed, combining the advantages of online delivery with the benefits of the school setting.

With regard to the content of mental health interventions, the strengthening of life skills is particularly recommended (WHO, 2021b). Such skills include communication, decision‐making, problem‐solving, and coping with stress and emotions. With the aim of coping with strain and promoting mental health, improving mental health literacy also has the potential to empower adolescents (Jorm, 2012; Ma, Anderson, & Burn, 2023). The topics are, for example, knowledge about mental health and mental illnesses, help‐seeking, prevention, and treatment (Ma et al., 2023).

Several studies have demonstrated the effectiveness of online interventions for the selective prevention of mental disorders in adolescent at‐risk populations (Clarke et al., 2015; Reitegger, Peras, Wright, & Gasteiger‐Klicpera, 2024). However, studies on universal online interventions to prevent the onset of mental disorders in mentally healthy adolescents are still rare (for reviews, see Clarke et al., 2015; Ebert, Cuijpers, Muñoz, & Baumeister, 2017; Noh & Kim, 2023). Clarke et al. (2015) reported on only one universal online intervention, which provided evidence for its effectiveness in relation to anxiety. This cognitive behavioral program designed to prevent anxiety and depression provided five interactive modules that could be accessed via a homepage and contained information, animations, quizzes, and homework exercises (Calear, Christensen, Mackinnon, Griffiths, & O'Kearney, 2009). When the *StresSOS* intervention was developed, to our knowledge, only one other study (Bannink et al., 2014) had reported positive results of universal online prevention in relation to general mental health. This intervention provided tailored feedback on one's own health behavior based on a screening result and recommendations for improving it.

Ebert et al. (2017) reported in their review only one universal online intervention, and it did not show a significant result. This intervention designed to prevent eating disorders consisted of five modules that could be accessed via a homepage (Lindenberg & Kordy, 2015). Recent meta‐analytic results (Noh & Kim, 2023) showed that online interventions in general adolescent populations for preventing anxiety, stress symptoms, or depression were only effective for depression. The studies that were examined mainly used cognitive behavioral therapy and family‐based interventions that were accessible via the internet. Reitegger et al. (2024) identified, among others, a modular structure, cognitive behavioral therapy approaches, brief psychoeducational elements, and gamification (e.g., quizzes, animations) as effective program components. Overall, studies (Clarke et al., 2015; Ebert et al., 2017; Noh & Kim, 2023) on the effectiveness of online preventive interventions have shown some promising results, but further research is urgently needed. Specifically, adequately powered RCTs using standard clinical diagnostic instruments and relevant outcome criteria (i.e., onset of mental disorders) are needed to advance the field (Ebert et al., 2017).

The current study was designed to evaluate the effectiveness of the newly developed universal school‐based internet intervention *StresSOS*, which addresses stress/coping and mental health literacy with the primary goal of reducing the proportion of adolescents who develop mental health problems over the course of a year. Based on frequently occurring mental health problems of youth (depressive symptoms, concerns about body weight, risky and addictive behavior; Solmi et al., 2022), a general mental health outcome was operationalized to identify and prevent the onset of mental health problems, as this was called for in relevant reviews (Ebert et al., 2017; Noh & Kim, 2023). We hypothesized that, compared with an active control group that received an internet‐based program about healthy nutrition, in the *StresSOS* group, significantly fewer participants would transition from the ‘healthy’ group to the status with impaired mental health at follow‐up about 1 year later.

As secondary outcomes, program adherence and program satisfaction were assessed as indicators of implementation. Reporting of participants' adherence to online interventions (i.e., the extent of program usage) remains inconsistent in the literature (e.g., Clarke et al., 2015; Noh & Kim, 2023) despite its potential importance for intervention effectiveness (e.g., Wright, Reitegger, Cela, Papst, & Gasteiger‐Klicpera, 2023). Based on Fridrici and Lohaus (2009), we hypothesized that approximately one third of participants would be compliant and complete the program, one third would utilize about half of the program, and one third would not engage with the program. To account for variation in adherence, the effectiveness of *StresSOS* was examined using both intention‐to‐treat and per‐protocol analyses. Program satisfaction was assessed because positive experiences in educational settings are associated with greater engagement and improved learning outcomes (e.g., King, McInerney, Ganotice, & Villarosa, 2015). Consistent with prior evidence from online prevention programs (Clarke et al., 2015), we expected high levels of program satisfaction among young people.

## Method

### Study design

A two‐arm, parallel‐group, randomized controlled trial that was stratified by sex was conducted. This trial was part of the ProHEAD project, a multicenter consortium investigating school‐based online mental health interventions (Kaess & Bauer, 2019). In the ProHEAD consortium, participants were assigned to one of five trials on the basis of their symptom status. The ProHEAD consortium included one universal mental health prevention program *StresSOS* (this trial; Eschenbeck et al., 2019) and four selective intervention studies for young people at risk of common mental disorders (Baldofski et al., 2019; Bauer et al., 2019; Diestelkamp et al., 2019; Kaess et al., 2019). Participants were recruited from secondary and vocational schools in five regions of Germany (Hamburg, Heidelberg, Leipzig, Marburg, Schwäbisch Gmünd).

The trial was preregistered with the German Register of Clinical Trials (DRKS00014693, https://drks.de/search/en/trial/DRKS00014693), and the study protocol was published (Eschenbeck et al., 2019; see Appendix S1 for the CONSORT checklist). Deviations from the study protocol are detailed in Appendix S2 (Table S1). To ensure that the study was conducted as planned and that the data were collected and secured correctly, a data and safety monitoring board was consulted annually.

### Participants

Permission to recruit in schools was granted by the federal authorities for all regions. If schools agreed to participate, classes between sixth grade and the final year were eligible to take part. Students who belonged to a participating class and were between 12 and 25 years old, with sufficient German language skills, their own e‐mail address, and access to the internet were invited to participate in the ProHEAD study. The invitation and information were provided by study staff as part of a presentation in class. Signed written informed consent (including parental consent for minors) was required to participate. If informed consent was given, participants completed a self‐report screening (there was no financial incentive for this). Age, sex, socioeconomic status, and mental health status were assessed. The assessment was web‐based and took place in the schools' computer labs. On the basis of the baseline screening results, participants were assigned to one of five trials (Kaess & Bauer, 2019). Students without mental health problems were eligible for the present trial (see the Primary Outcome section for further details).

The primary endpoint was also assessed on site in the schools 12 months after recruitment. Due to school closures caused by the COVID‐19 pandemic during the project, it was no longer possible to conduct assessments in schools. However, after ethics approval, follow‐ups were conducted online throughout the school lockdowns. In this case, students received an e‐mail with a link to complete the follow‐up assessment outside school. As a consequence of the pandemic, rates of missing data at follow‐up were elevated.

### Randomization and masking

After the school‐based screening, eligible students were invited to take part in the *StresSOS* trial by e‐mail. Participants received trial information and an activation link and could access the program with a user name and password of their choice. Those who activated their account were randomly allocated to either the intervention group (IG; *StresSOS*) or the attention placebo control group (CG; Healthy nutrition; 1:1 ratio) stratified by sex. The randomization was conducted by computer and based on a permutated block design with variable, randomly arranged block sizes. Participants were not told which study arm was ‘the intervention of interest’. The data were collected with online questionnaires. The group allocation was not masked for data analyses.

### Procedures

The conditions were structurally equivalent, and the control condition replicated as many components of the *StresSOS* intervention as possible. Both programs were offered via the internet and could be used by the students at home in their free time ad libitum. In both conditions, participants had the opportunity to take part in eight fully automated sessions, which were activated week by week. The sessions consisted of an information component with text passages, videos, and audio files, followed by an exercise section on the respective topic. For each session, weekly homework was given to support transfer to everyday life. Once per week, participants received short fully automated teasers about the content of the next session via e‐mail as reminders to take part in the program and to provide feedback on whether they had completed the homework and whether they found it helpful (monitoring). In addition, in the *StresSOS* condition, group chats were offered twice per week by an advanced student from the field of education or health promotion.

The *StresSOS* program was based on approaches to life skills (WHO, 2021b), especially stress/coping, and research on mental health literacy (Wei, Hayden, Kutcher, Zygmunt, & McGrath, 2013). The content of the control condition (Healthy Nutrition Program) was based on recommendations from the German Nutrition Society (2015); for further information, see Appendix S3 and Eschenbeck et al. (2019).

### Measures

#### Primary outcome

Mental health status as the primary outcome was assessed at baseline and 12 months after randomization. The primary outcome was the prevention of mental health deterioration, operationalized by a transition to the at‐risk or severe symptoms group in accordance with the screening and predefined assignment criteria from the ProHEAD consortium (Kaess & Bauer, 2019).

Mental health status (i.e., without mental health problems vs. at risk or with mental health problems) was assessed as a composite with a set of validated questionnaires that were clinically well‐established and were able to identify the onset of mental health problems (Eschenbeck et al., 2019; Kaess & Bauer, 2019). We used the *Strengths and Difficulties Questionnaire* (SDQ; score 0–40; Goodman & Goodman, 2009) to measure broad psychopathology; the *Short Evaluation of Eating Disorders* (SEED) to calculate body mass index (BMI) and to evaluate fear of weight gain (Bauer, Winn, Schmidt, & Kordy, 2005); the *Weight Concerns Scale* (WCS; score 0–100; Killen et al., 1994) to assess concerns about body weight; the *Car, Relax, Alone, Forget, Friends, Trouble questionnaire* (CRAFFT‐d; score 0–6; Tossmann, Kasten, Lang, & Strüber, 2009) to assess problematic substance use; the *Alcohol Use Disorders Identification Test* (AUDIT; score 0–40; Babor, Higgins‐Biddle, Saunders, & Monteiro, 2001) to investigate hazardous alcohol consumption; and the *Patient Health Questionnaire‐9 modified for Adolescents* (PHQ‐A; score 0–27; Johnson, Harris, Spitzer, & Williams, 2002) to screen for depressive symptoms and suicidality.

Once 10% of the ProHEAD target sample was reached, a preliminary data analysis was conducted to evaluate the preregistered allocation ratio, and the ratio was adjusted. Individuals with at least one value above the following cut‐offs were assigned to the group as having impaired mental health (i.e., ‘at risk or with mental health problems’, Figure S1 in Appendix S4): SDQ ≥20, WCS ≥58, BMI <5th age/sex percentile with a coexisting fear of weight gain (SEED), CRAFFT‐d ≥2, AUDIT ≥20, PHQ‐A ≥10, or suicidal tendencies. Participants with all values below the specified thresholds were assigned to the ‘without mental health problems’ group (Eschenbeck et al., 2019; Kaess & Bauer, 2019).

#### Secondary outcomes

As secondary outcomes, program adherence and program satisfaction were investigated (Eschenbeck et al., 2019). Adherence in the sessions was evaluated using log data. A session was considered ‘attended’ if the information section was opened (as most of the intervention content was on this page), regardless of whether the exercise section had been completed. The responses to the monitoring questions were evaluated with log data. The participants were asked whether they had done the homework and whether they liked it (dichotomous yes/no response format each). Program satisfaction was operationalized through the German version of the *Patient Satisfaction Questionnaire* (ZUF‐8; Schmidt, Lamprecht, & Wittmann, 1989) adapted to the internet‐based setting and the prevention context (score 8–32; higher scores indicate higher program satisfaction). It was assessed with the 12‐month follow‐up survey.

### Power and sample size

We expected 30% of the participants to transition from ‘without mental health problems’ to ‘at risk or with mental health problems’ in the CG compared with 15% in the IG. Thus, participation in *StresSOS* should prevent 50% of the transitions to impaired mental health. In order to detect the expected effect with a power of 95% at the 12‐month follow‐up, a sample size of *n* = 418 was required (Fisher's exact test; two‐sided, *α* = .05). See Eschenbeck et al. (2019) for a comprehensive description of the sample size calculation for this study and the non‐inferiority trial (Study B).

### Statistical analysis

Fisher's exact test (two‐sided, *α* = .05) was used to determine differences between the IG and CG in the primary outcome. The main analyses were performed in accordance with the intention‐to‐treat principles, that is, all students who were randomly assigned and participated in the follow‐up survey were analyzed, regardless of whether the program was used. In addition, per‐protocol analyses were conducted. All students who utilized at least one session and one monitoring questionnaire were included in the per‐protocol analyses.

Program adherence was analyzed using the program logs. Group differences were analyzed with a chi‐square test. Group differences in program satisfaction were examined using a two‐sided *t*‐test (*α* = .05). To illustrate the results, the items were dichotomized and presented as percentages of mostly/very satisfied or mostly/very dissatisfied.

Due to loss to follow‐up of 52%, a complete case analysis was conducted and we did not perform multiple imputation. This is because the high rate of missing data can severely impair the stability and validity of the imputation results (Rubin, 1987). For nonresponse analysis, logistic regression with sociodemographics as control variables (Table S2), and exploratory group comparisons as a function of adherence (Table S3), see Appendix S5. The program R was used for randomization (Version 3.5.1) and data analyses (Version 4.3.2).

## Results

**Figure 1:**
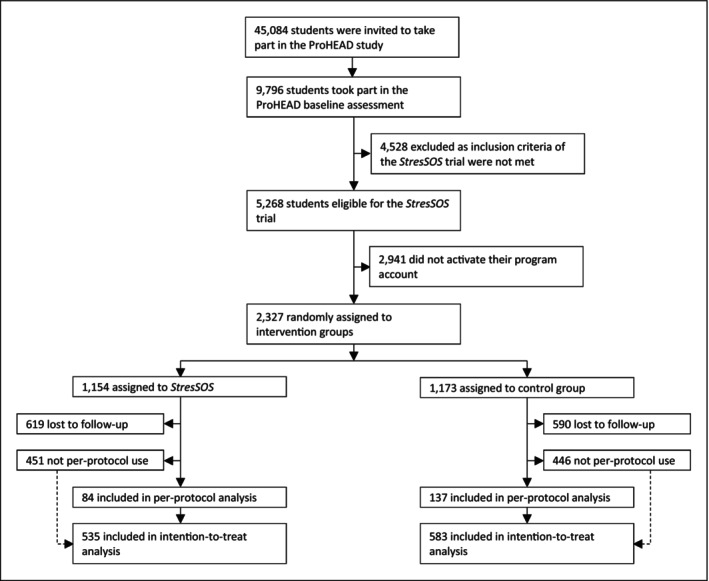
*StresSOS* trial profile

Out of 767 invited schools, 195 (25%) agreed to take part in the study. Between Dec 11, 2018, and Feb 14, 2022, of the 45,084 students who had been invited to participate, 9,796 students participated in the ProHEAD baseline screening, of whom 5,268 were eligible for the *StresSOS* trial. Of these, 2,941 (56%) did not activate their account. A total of 2,327 (44%) students were randomly assigned to either the *StresSOS* condition (*n* = 1,154) or the CG (*n* = 1,173), with 1,209 (52%) of the randomized participants lost to follow‐up (619 in the IG, 590 in the CG), mainly due to COVID‐19‐related school lockdowns. Forty‐eight percent of the randomized participants (1,118 students) completed their follow‐up and were included in the intention‐to‐treat analyses (primary endpoint), 535 in the IG, and 583 in the CG. Of those, 221 (20%) utilized at least one session and one monitoring questionnaire and were included in the per‐protocol analysis (84 in the IG, 137 in the CG; Figure 1). The final participant completed the 12‐month follow‐up on Dec 31, 2022.

### Participant characteristics

Table 1 presents the baseline characteristics. Participants had a mean age of *M* = 14.6 years (*SD* = 1.9, Range: 12–24 years), and the majority were female (701 [63%] of 1,118), stated that they came from families with a high socioeconomic status (906 [81%]), and attended gymnasium (secondary school leading to higher education/university entrance qualification; 783 [70%]). Between the complete cases (*n* = 1,118) and dropouts (*n* = 1,209) a small and statistically significant difference was observed for age (*d* = 0.1), sex (*V* = 0.06), and type of school (*V* = 0.07), but not for mental health status (Appendix S1).

**Table 1:** Participant characteristics at baseline (*N* = 1,118)

	*StresSOS* (*n* = 535)	Control (*n* = 583)
Age, mean (*SD*)	14.47 (1.82)	14.66 (1.91)
Children (12 years), *n* (%)	64 (11.96)	63 (10.81)
Adolescents (13–17 years), *n* (%)	446 (83.36)	477 (81.82)
Young adults (18–24 years), *n* (%)	25 (4.67)	43 (7.38)
Sex, *n* (%)		
Female	331 (61.87)	370 (63.46)
Male	204 (38.13)	213 (36.54)
Socioeconomic status^a^, *n* (%)		
Low/medium	90 (16.82)	122 (20.93)
High	445 (83.18)	461 (79.07)
School type^b^, *n* (%)		
Others	155 (29.03)	178 (30.58)
Gymnasium	379 (70.97)	404 (69.42)

^a^ Socioeconomic status was recorded with the *Family Affluence Scale* (Boyce, Torsheim, Currie, & Zambon, 2006).

^b^ No information on the school type was available for one person per group. Other school types: secondary schools leading to the lower or intermediate school leaving certificate or to the completion of vocational training. Gymnasium (secondary school leading to higher education/university entrance qualification).

#### Primary outcome

At the 12‐month follow‐up, the *StresSOS* IG significantly prevented more transitions to the ‘at risk or with mental health problems’ group than the CG (Table 2). The per‐protocol analysis showed larger effects in the same direction with significantly fewer individuals transitioning to the ‘at risk or with mental health problems’ group in the IG than in the CG (Table 3). See Appendix S6 for separate analyses of the different measures used to create the combined outcome mental health status (Table S4).

**Table 2:** Intention‐to‐treat analysis of mental health status in the *StresSOS* and control groups at baseline and at the 12‐month follow‐up

	*StresSOS*	Control	OR	95% CI	*p*
Baseline					
Without MHP, *n* (%)	535 (100)	583 (100)			
Follow‐up					
Without MHP, *n* (%)	422 (79)	421 (72)	0.70	[0.52, 0.92]	<.05
At risk or with MHP, *n* (%)	113 (21)	162 (28)			

MHP, mental health problems.

**Table 3:** Per‐protocol analysis of mental health status in the *StresSOS* and control groups at baseline and at the 12‐month follow‐up

	*StresSOS*	Control	OR	95% CI	*p*
Baseline					
Without MHP, *n* (%)	84 (100)	137 (100)			
Follow‐up					
Without MHP, *n* (%)	72 (86)	96 (70)	0.39	[0.17, 0.83]	< .01
At risk or with MHP, *n* (%)	12 (14)	41 (30)			

MHP, mental health problems.

Controlling for sociodemographic variables, the logistic regression revealed that the significant effect of group on mental health status remained (Appendix S5).

#### Secondary outcomes

About 30% (*n* = 159) of the IG and 44% (*n* = 257) of the CG used the program sessions. There was a difference in session participation between the two groups, *χ* ^2^(3) = 42.20, *p* < .001 (Table 4). Almost 40% of participants in both groups answered the monitoring questions. There was no difference in the frequencies of the monitoring responses between the IG and the CG, *χ* ^2^(3) = 3.66, *p* = .30 (Table 4). Across all monitoring surveys relating to the various sessions, the majority of respondents agreed with the question of whether they liked the homework: In the IG, 268 out of 414 answers (65%) were positive, and in the CG, it was 363 out of 499 (73%). Across the entire study period, the group chat was used by one user for one appointment. The content of the chat referred to a dispute between friends and how to resolve it.

**Table 4:** Adherence in the programs

	Session participation, *n* (%)		Monitoring response, *n* (%)	
	*StresSOS*	Control	*StresSOS*	Control
Once	101 (18.88)	149 (25.56)	106 (19.81)	103 (17.67)
2 to 5 times	48 (8.97)	53 (9.09)	85 (15.89)	88 (15.09)
≥ 6 times	10 (1.87)	55 (9.43)	11 (2.06)	12 (3.77)
Never	376 (70.28)	326 (55.92)	333 (62.24)	370 (63.46)

Program satisfaction was high (*M* = 22.49, *SD* = 3.92, *n* = 1,098). There was no difference between the IG (*M* = 22.55, *SD* = 3.68, *n* = 527) and the CG (*M* = 22.42, *SD* = 4.13, *n* = 571), *t*(1096) = 0.55, *p* = .58. When the IG and the CG were combined, 56% of the participants were satisfied with the program, 75% agreed that the program met their individual needs, 88% would use the program again, and 93% were satisfied with the level of help they received (Table 5). No adverse events attributable to *StresSOS* or the control condition were reported.

**Table 5:** Participant satisfaction with the programs

	*StresSOS*	Control	Total
	(*n* = 527)	(*n* = 571)	(*N* = 1,098)
1. The program was of good quality	63.9%	63.6%	63.8%
2. I received the kind of support I wanted	72.9%	72.9%	72.9%
3. The program has met most or all of my needs	73.6%	76.0%	74.9%
4. I would recommend the program to my friends	63.0%	63.9%	63.5%
5. I am satisfied with the amount of help I have received	93.9%	93.0%	93.4%
6. The program has helped me to deal with my problems more appropriately	69.4%	68.1%	68.8%
7. On the whole, I am satisfied with the program	56.0%	55.2%	55.6%
8. I would use the program again	88.0%	88.4%	88.3%

Percentage consent mostly/very satisfied.

## Discussion

This large‐scale RCT showed that the school‐based internet intervention *StresSOS*, combining life skills approaches focusing on stress/coping with content about mental health literacy, was able to significantly reduce the incidence rate of mental health problems in young people compared with the control intervention within 1 year. Whereas most previous research on online prevention approaches focused on at‐risk or clinical samples (Clarke et al., 2015; Noh & Kim, 2023; Reitegger et al., 2024), this study is one of the first adequately powered RCTs in the field of universal online prevention for common mental illnesses in young people without mental health problems. The observed proportion of 28% in the CG that showed deteriorations in mental health (transitions from ‘without mental health problems’ to ‘at risk or with mental health problems’), as the primary outcome, corresponds well with the preregistered estimate (Eschenbeck et al., 2019). In the *StresSOS* condition, these transitions to worse mental health were prevented by one quarter in the intention‐to‐treat analyses (small effect) and by half in the per‐protocol analyses (medium effect). Halving the rate to worse mental health corresponds with the preregistered estimate (Eschenbeck et al., 2019). However, it required a minimum level of program adherence (see Wright et al., 2023). The program content on stress/coping (unlike, perhaps, mental health and mental illness in the penultimate, rarely used session) may have fostered a helpful ‘neutral attitude’ toward mental health (see Ndour & Foulkes, 2025).

Although low adherence is a well‐known challenge of online interventions (Clarke et al., 2015), program use was even lower than expected (Fridrici & Lohaus, 2009). Reasons for low adherence were not assessed. However, it is plausible to assume that many adolescents did not see a need to engage with an intervention or did not consider it a priority because they had not experienced any mental health problems at baseline. Others may have had no interest in the topic, lost interest in it (Steiner & Gest, 1996), or may have had no time apart from school. This would also align with results for nonparticipation in the ProHEAD baseline, which showed that lack of interest and lack of time were among the main reasons (Baldofski et al., 2024). The observation that the participants in the CG utilized the program on healthy nutrition slightly more than the IG participants used the mental health program could be understood through results of a study (Steiner & Gest, 1996) that found that less than 20% of healthy adolescents liked to talk about mental health issues, whereas more than 50% liked to talk about nutrition. As the per‐protocol analysis in this study indicates, it has also been previously shown that greater adherence is associated with better outcomes (for online stress prevention; e.g., Fridrici & Lohaus, 2009). However, one study (females only) found no difference in the reduction in depressive symptoms between participants with low and high adherence (O'Kearney, Kang, Christensen, & Griffiths, 2009). Further research is needed to clarify the subgroups for which it might be particularly important to strengthen program adherence. For example, are they those who show particularly low adherence (in our study, boys and participants with low/medium socioeconomic status) and/or those who are more vulnerable to developing mental illness (in our study, girls and older adolescents)?

In line with our expectations, program satisfaction was moderate to high. This finding has frequently been reported in studies evaluating online mental health promotion and prevention programs (Clarke et al., 2015). The fact that the participants were fully respected in their autonomy and could decide independently whether and to what extent they wanted to use the program, corresponding with adolescents' need for self‐determination, may have had a positive impact on the evaluation (e.g., Eagleton, Williams, & Merten, 2016). The online program may also have benefited to participate ‘privately’ without social evaluations of self‐disclosures by classmates (Foulkes & Andrews, 2025).

The strengths of this trial include the study design as an RCT with the implementation of an active attention placebo CG, an evaluation that was conducted in accordance with intention‐to‐treat principles, a large sample size, nationwide recruitment across all school types in the ProHEAD project, and the use of a relatively broad age range. In addition, validated, international questionnaires were used to assess mental health problems, and the categorization criteria within the study were adapted to prevalence rates. With regard to the *StresSOS* program, the selection of content was theory‐based and corresponds to the current recommendations of the WHO (2021a). Despite its strengths, limitations also need to be acknowledged. First, the loss to follow‐up rate of 52% was high and led to slightly larger proportions of girls, younger students, and students in higher education. However, this rate applied equally to the CG and IG, and the intervention effect remained when controlling for the sociodemographic variables. Nevertheless, the generalizability of our findings is limited and can only be concluded with certainty for the population studied. Second, the fact that minors required parental consent could have led to a selection bias; younger, higher educated students may have been overrepresented (Shaw, Cross, Thomas, & Zubrick, 2014). Although we realized a high recruitment effort and tried to formulate the information for parents inclusively, future studies could focus on diversity‐sensitive strategies to increase the participation rate. The challenge of declining willingness to participate in scientific studies was also highlighted in other large‐scale school‐based studies (Winter et al., 2024), accompanied by the call for the implementation of incentive structures. Third, data collection was based on self‐report and not on clinical interviews. Even if the instruments were adapted for adolescents, it is possible that instructions or content were misunderstood. However, young school‐age children were able to provide accurate information about their own health (Riley, 2004), and the questionnaires that were used are well‐validated instruments for use as self‐report methods.

## Conclusion

This study demonstrates the benefits and potential of the school‐based *StresSOS* program for the universal prevention of mental health problems in young people, with a small (intention‐to‐treat) to medium effect (per‐protocol use). The broad and sustained provision of the intervention could contribute to a substantial reduction in the burden of disease on the population level. The internet‐based format and utilization of mostly automated components would allow for the intervention of large samples at low costs (Buntrock et al., 2017) and with little personnel effort. Implementation in school contexts is recommended, as this approach ensures that entire cohorts are addressed. The promising findings of the present study may lay the foundation for subsequent research related to the *StresSOS* program (e.g., health economic evaluation implementation research) and may inform other studies related to online health promotion and prevention programs, ultimately serving the socioemotional development and well‐being of individuals.

## Trial registration

The trial was preregistered with the German Register of Clinical Trials (DRKS00014693, date of registration: May 14, 2018, see https://drks.de/search/en/trial/DRKS00014693).

## Ethical considerations

The study was approved by the ethics committee of the leading study site, the Medical Faculty Heidelberg (Study‐ID: S‐086/2018, date of approval: March 1, 2018). Signed written informed consent was required to participate, and in the case of minors, signed written parental consent was also obtained.

**Key points**

**What's known?**

- Evidence concerning the effectiveness of internet interventions for the universal prevention of mental illness among healthy young people is inconclusive.

**What's new?**

- This research is a large‐scale RCT with the implementation of an active attention placebo control group, an evaluation that was in accordance with intention‐to‐treat principles, a large sample size, and validated clinically established instruments.
- Results confirm the effectiveness of the school‐based internet intervention, which combined life skills approaches with content about mental health literacy, in the universal prevention of mental health problems in young individuals.

**What's relevant?**

- Continued evaluation and implementation into mental health prevention practice in schools is recommended to significantly contribute to the prevention of mental illnesses and reduce the high burden of disease at the population level.

## Supporting information


**Appendix S1.** CONSORT 2010 checklist.
**Appendix S2.** Deviations from the study protocol.
**Table S1.** Deviations from the study protocol with reasons and consequences.
**Appendix S3.** Program content.
**Appendix S4.** Group assignment for the primary outcome.
**Appendix S5.** Nonresponse analysis, logistic regression, and exploratory group comparison.
**Table S2.** Regression results for mental health status at the 12‐month follow‐up.
**Table S3.** Comparisons between groups (*N* = 535).
**Appendix S6.** Fisher's exact test separately for the different measures used to create the primary outcome.
**Table S4.** Fisher's exact test for each measure of the combined primary outcome mental health status at the 12‐month follow‐up.

**Figure S1.** Group assignment for the primary outcome.

## Data Availability

Data sharing is restricted because participant consent did not include provisions for data dissemination beyond the original research objectives, in accordance with applicable ethical and legal requirements. The consent forms and patient information sheets are available for 5 years after publication of the article at https://www.prohead.de/zentral/infoschreiben.
