# Supplementary material for: Effectiveness of the school‐based internet intervention StresSOS for the prevention of mental health problems in young people: a randomized controlled trial as part of the ProHEAD consortium
Source: J Child Psychol Psychiatry. 2026 Mar 13;67(8):1393–403. doi: 10.1111/jcpp.70145 (PMC13341385; doi:10.1111/jcpp.70145)
Supplement: Supplementary file 1 — Appendix S1. CONSORT 2010 checklist. Appendix S2. Deviations from the study protocol. Table S1. Deviations from the study protocol with reasons and consequences. Appendix S3. Program content. Appendix S4. Group assignment for the primary outcome. Appendix S5. Nonresponse analysis, logistic regression, and exploratory group comparison. Table S2. Regression results for mental health status at the 12‐month follow‐up. Table S3. Comparisons between groups (N = 535). Appendix S6. Fisher's exact test separately for the different measures used to create the primary outcome. Table S4. Fisher's exact test for each measure of the combined primary outcome mental health status at the 12‐month follow‐up. [file JCPP-67-1393-s001.docx]

## Effectiveness of the School-Based Internet Intervention *StresSOS* for the Prevention of Mental Health Problems in Young People: A Randomized Controlled Trial as Part of the ProHEAD Consortium

**Supporting Information**

**
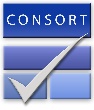
 Appendix S1. CONSORT 2010 checklist of information to include when reporting a randomised trial***

| **Section/Topic** | **Item No** | **Checklist item** | **Reported on page No** |
| --- | --- | --- | --- |
| **Title and abstract** | | | |
|  | 1a | Identification as a randomised trial in the title | 1 |
|  | 1b | Structured summary of trial design, methods, results, and conclusions (for specific guidance see CONSORT for abstracts) | 3 |
| **Introduction** | | | |
| Background and objectives | 2a | Scientific background and explanation of rationale | 5-8 |
|  | 2b | Specific objectives or hypotheses | 7-8 |
| **Methods** | | | |
| Trial design | 3a | Description of trial design (such as parallel, factorial) including allocation ratio | 8-9 |
|  | 3b | Important changes to methods after trial commencement (such as eligibility criteria), with reasons | 9-10,  Appendix S2 |
| Participants | 4a | Eligibility criteria for participants | 9, 11-12 |
|  | 4b | Settings and locations where the data were collected | 8 |
| Interventions | 5 | The interventions for each group with sufficient details to allow replication, including how and when they were actually administered | 10,  Appendix S3 |
| Outcomes | 6a | Completely defined pre-specified primary and secondary outcome measures, including how and when they were assessed | 11-12 |
|  | 6b | Any changes to trial outcomes after the trial commenced, with reasons | Appendix S2 |
| Sample size | 7a | How sample size was determined | 12 |
|  | 7b | When applicable, explanation of any interim analyses and stopping guidelines |  |
| Randomisation: |  |  |  |
| Sequence generation | 8a | Method used to generate the random allocation sequence | 10-11 |
|  | 8b | Type of randomisation; details of any restriction (such as blocking and block size) | 10-11 |
| Allocation concealment mechanism | 9 | Mechanism used to implement the random allocation sequence (such as sequentially numbered containers), describing any steps taken to conceal the sequence until interventions were assigned | 10-11 |
| Implementation | 10 | Who generated the random allocation sequence, who enrolled participants, and who assigned participants to interventions | 10-11 |
| Blinding | 11a | If done, who was blinded after assignment to interventions (for example, participants, care providers, those assessing outcomes) and how |  |
|  | 11b | If relevant, description of the similarity of interventions | 10,  Appendix S3 |
| Statistical methods | 12a | Statistical methods used to compare groups for primary and secondary outcomes | 12-13 |
|  | 12b | Methods for additional analyses, such as subgroup analyses and adjusted analyses | 12-13,  AppendixS5, Appendix S6 |
| **Results** | | | |
| Participant flow (a diagram is strongly recommended) | 13a | For each group, the numbers of participants who were randomly assigned, received intended treatment, and were analysed for the primary outcome | 13-14 |
|  | 13b | For each group, losses and exclusions after randomisation, together with reasons | 13-14 |
| Recruitment | 14a | Dates defining the periods of recruitment and follow-up | 13-14 |
|  | 14b | Why the trial ended or was stopped | 13-14 |
| Baseline data | 15 | A table showing baseline demographic and clinical characteristics for each group | 14, Table 1 |
| Numbers analysed | 16 | For each group, number of participants (denominator) included in each analysis and whether the analysis was by original assigned groups | 13-14 |
| Outcomes and estimation | 17a | For each primary and secondary outcome, results for each group, and the estimated effect size and its precision (such as 95% confidence interval) | 13-15,  Table 2,  Table 3,  Table 4,  Table 5 |
|  | 17b | For binary outcomes, presentation of both absolute and relative effect sizes is recommended | Table 2,  Table 3 |
| Ancillary analyses | 18 | Results of any other analyses performed, including subgroup analyses and adjusted analyses, distinguishing pre-specified from exploratory | AppendixS5, Appendix S6 |
| Harms | 19 | All important harms or unintended effects in each group (for specific guidance see CONSORT for harms) |  |
| **Discussion** | | | |
| Limitations | 20 | Trial limitations, addressing sources of potential bias, imprecision, and, if relevant, multiplicity of analyses | 17-18 |
| Generalisability | 21 | Generalisability (external validity, applicability) of the trial findings | 17-18 |
| Interpretation | 22 | Interpretation consistent with results, balancing benefits and harms, and considering other relevant evidence | 18 |
| **Other information** | | |  |
| Registration | 23 | Registration number and name of trial registry | 3, 8 |
| Protocol | 24 | Where the full trial protocol can be accessed, if available | 8 |
| Funding | 25 | Sources of funding and other support (such as supply of drugs), role of funders | Appendix S7 |

Citation: Schulz KF, Altman DG, Moher D, for the CONSORT Group. CONSORT 2010 Statement: updated guidelines for reporting parallel group randomised trials. BMC Medicine. 2010;8:18.
© 2010 Schulz et al. This is an Open Access article distributed under the terms of the Creative Commons Attribution License (<http://creativecommons.org/licenses/by/2.0>), which permits unrestricted use, distribution, and reproduction in any medium, provided the original work is properly cited.

*We strongly recommend reading this statement in conjunction with the CONSORT 2010 Explanation and Elaboration for important clarifications on all the items. If relevant, we also recommend reading CONSORT extensions for cluster randomised trials, non-inferiority and equivalence trials, non-pharmacological treatments, herbal interventions, and pragmatic trials. Additional extensions are forthcoming: for those and for up-to-date references relevant to this checklist, see [www.consort-statement.org](http://www.consort-statement.org).

# Appendix S2

## Table S1. Deviations from the Study Protocol with Reasons and Consequences

| **Analyses** | **Planned (yes/no)** | **Performed (yes/no)** | **Reasons** | **Consequences** |
| --- | --- | --- | --- | --- |
| **Primary outcome at the 12-month follow-up** |  |  |  |  |
| Fisher’s exact test based on intention to treat principles | Yes | Yes | - | - |
| Predictive mean matching to impute missing values | Yes | No | Multiple imputation was not performed to avoid that the high rate of missing data severely impairs the stability and validity of the results (Rubin, 1987). | Complete case analysis performed. |
| Fisher’s exact test based on per-protocol principles | No | Yes | Since adherence was lower than expected, additional per-protocol analyses were performed to obtain an approximate estimate of the effect of the program under ideal conditions. | Additional analyses performed. |
| Fisher's exact test calculated separately for the different measures used to create the primary outcome mental health status | No | Yes | To assess the intervention effect separately for the different measures used to create the primary outcome mental health status, the analyses were additionally carried out separately for each measure using previously defined assignment criteria (without mental health problems vs. at risk or with mental health problems). | Separate analyses performed. |
| Logistic regression with age, sex, school type, and socioeconomic status | No | Yes | Given that age, sex, and school type differed between the participants who were lost to follow-up and those who participated, a logistic regression was performed to test whether the intervention effect remained after controlling for these variables. Socioeconomic status was also included, as associations with mental health outcomes are documented. Most of the participants came from families with a high socioeconomic status, compared with participants from families with low/middle socioeconomic status. | Logistic regression performed to control for sociodemographic variables. |
| **Secondary outcomes** |  |  |  |  |
| Chi-square test for group differences in program adherence | Yes | Yes | - | - |
| *t-*test to analyze group differences in program satisfaction | Yes | Yes | - | - |
| Repeated-measures analysis of variance and covariance related to stress/coping and mental health literacy | Yes | No | The assessment of these variables was less successful than expected. Data were collected in a trial specific baseline (*StresSOS*: *n* = 535, control group: *n* = 583) and post intervention (*StresSOS*: *n* = 124 [23%], control group: *n* = 142 [24%]), separate from the school assessments. The low response rate hindered the planned analyses, and neither complete case analysis nor data imputation adequately addressed the issue. | Analysis not performed. |
| Group comparisons as a function of program adherence | No | Yes | In response to a reviewer's comment, we post-hoc explored whether there are subgroups that show especially low program adherence. Among the *StresSOS* intervention group (*n* = 535), comparisons (Chi-square test, *t*-test) between participants who utilized at least one *StresSOS* session and one monitoring questionnaire (per-protocol subsample, *n* = 84; indicating higher adherence) and those who did not use at least one *StresSOS* session and one monitoring (intention-to-treat sample without per-protocol subsample, *n* = 451; indicating lower adherence) were performed with regard to the sociodemographic variables age, sex, socioeconomic status, and school type. | Additional analyses performed. |
| **Analyses at the 24-month follow-up** | Yes | No | Within the ProHEAD project data was assessed during two follow-ups at 1 and 2 years after baseline. The ProHEAD project encompassed four other trials besides the *StresSOS* trial (Kaess & Bauer, 2019). The other trials focused on adolescents with risk for mental health problems or with clinically relevant mental health problems. To adhere to ethical standards and at the same time booster recruitment for the other RCTs of the ProHEAD consortium, participants from the *StresSOS* trial, who presented at least at-risk for mental health problems at the 1-year follow-up (primary endpoint of the *StresSOS* RCT), were invited to participate in one of the other clinical trials that intended to either provide indicated prevention or support for professional help-seeking (if they met the inclusion criteria for these trials at that time). As a consequence, all participants of the *StresSOS* trial that transitioned to the “at risk or with mental health problems” group were invited to participate in another trial. As a result, the 24-month follow-up data would be systematically biased because participants were offered participation in other interventions when they displayed risk. Participation in *StresSOS* after Follow-up 1 was not permitted. | Analysis not performed. |

References

Kaess, M., & Bauer, S. (2019). Editorial Promoting Help-seeking using E-Technology for ADolescents: The ProHEAD consortium. *Trials, 20*(1), 72.

Rubin, D. B. (1987). *Multiple Imputation for Nonresponse in Surveys.* New York: Wiley.

# Appendix S3

## Detailed Information on Program Content

The *StresSOS* program is based on approaches to life skills (WHO, 2021), especially stress/coping, and research on mental health literacy (Wei, Hayden, Kutcher, Zygmunt, & McGrath, 2013). Accordingly, the *StresSOS* program provides knowledge about coping skills (problem solving, cognitive reconstruction, relaxation, seeking support and the relationships between thoughts, feelings, and behaviors). It also provides information about mental health/illness and help-seeking. Detailed content of the modules is described elsewhere (Eschenbeck et al., 2019). *StresSOS* was initially developed as a face-to-face program (Lehner et al., 2022) at the Schwäbisch Gmünd site. After pretesting in four classes, the first version was revised and adapted. In a second step, the program was translated into an internet-based format with technical support coming from the study center in Heidelberg, Germany.

The content of the control condition (Healthy Nutrition Program) is based on recommendations from the German Society for Nutrition about healthy nutrition (for further information, see Eschenbeck et al., 2019). It was developed at the Schwäbisch Gmünd site in consultation with an oecotrophologist from the German Nutrition Society. Care was taken to implement the program structurally as parallel as possible to the *StresSOS* program (i.e., it comprised the same number of sessions and the same overall page structure) but not to create any proximity in terms of content. In order to reduce the possibility of expectation effects, care was taken to choose a topic from the field of health promotion, which is just as plausible an intervention for mental health for adolescents as the the “intervention of interest”.

References

Eschenbeck, H., Lehner, L., Hofmann, H., Bauer, S., Becker, K., Diestelkamp, S., Kaess, M., Moessner, M., Rummel-Kluge, C., Salize, H.-J., & the ProHEAD Consortium (2019). School-based mental health promotion in children and adolescents with StresSOS using online or face-to-face interventions: Study protocol for a randomized controlled trial within the ProHEAD consortium. *Trials, 20*(1), 64.

Lehner, L., Gillé, V., Baldofski, S., Bauer, S., Becker, K., Diestelkamp, S., Kaess, M., Krämer, K., Lustig, S., Moessner, M., Rummel-Kluge, C., Thomasius, R., Eschenbeck, H., & the ProHEAD Consortium (2022). Moderators of pre-post changes in school-based mental health promotion: Psychological stress symptom decrease for adolescents with mental health problems, knowledge increase for all. *Frontiers in Psychiatry, 13*, 899185.

Wei, Y., Hayden, J. A., Kutcher, S., Zygmunt, A., & McGrath, P. (2013). The effectiveness of school mental health literacy programs to address knowledge, attitudes and help seeking among youth. *Early Intervention in Psychiatry, 7*(2), 109–121.

WHO (2021). *Mental health in schools: A manual.* Cairo: WHO Regional Office for the Eastern Mediterranean.

# Appendix S4

**Group Assignment for the Primary Outcome**

# Uploaded in a separate file

# Appendix S5

## Nonresponse Analysis

To assess the pattern of loss to follow-up, we compared the baseline values on sociodemographic variables and other characteristics between complete cases and incomplete cases. Chi-square tests were calculated for categorical data and independent *t*-tests were calculated for continuous data (two-sided, α = .05).

Comparisons between complete cases (*n* = 1,118) and dropouts (*n* = 1,209) revealed no statistically significant differences for the measures used to calculate the primary outcome (mental health status), emotional and behavioral problems (SDQ; *p* = .90), weight and shape concerns (WCS; *p* = .86), hazardous alcohol consumption (AUDIT; *p* = .07), and depressive symptoms and suicidality (PHQ-A; *p* = .39) at baseline.

For the sociodemographic variables, there was no significant difference for socioeconomic status (*p* = .19). A small difference was observed for age, *t*(2312.8) = 2.83, *p* < .01, Cohen’s *d* = 0.1, and for sex, χ²(1) = 8.56, *p* < .01, Cramer’s *V* = 0.06. Complete cases were slightly younger (14.57 years, *SD* = 1.87) than dropouts (14.81 years, *SD* = 2.17), and there was a larger proportion of females with complete cases (62.7%) compared with dropouts (56.7%). The distribution of school types also differed with a small effect size, χ²(1) = 12.51, *p* < .001, Cramer’s *V* = 0.07, with more grammar school students among the complete cases (70.2%) than among the dropouts (63.1%).

When interpreting these results, it is necessary to consider that, although statistically significant differences were found between the complete cases and the cases with missing data for age (*d* = 0.1), sex (*V* = 0.06), and type of school (*V* = 0.07), these differences could be assumed to have only small practical relevance.

**Logistic Regression With Sociodemographic Variables as Control Variables**

A logistic regression analysis was calculated on mental health status at the 12-month follow up as outcome to test whether the effect of group (IG, CG) remained if participant’s sociodemographic variables were included as predictors. The following predictor variables, for which there were small differences between complete cases and dropouts (see Nonresponse Analysis) and/or associations with mental health outcomes, were taken into account: Age (e.g., Kieling et al, 2024; Solmi et al., 2022), sex (e.g., Kieling et al, 2024), school type (e.g., Evensen, 2019, for student achievement in schools), and socioeconomic status (e.g., Klipker et al., 2018). All five predictors (group, age, sex, school type, socioeconomic status) were entered into the model. Due to two cases with missing values on school type, the analysis is based on data from 1,116 participants (534 in the IG, 582 in the CG; 274 at risk or with mental health problems, 842 without mental health problems).

The model explained about 2.7% of the variance in the outcome (Nagelkerke *R*² = 0.027), χ²(5) = 20.72, *p* < .001. Results are shown in Table S2. The predictor group showed a significant positive effect on the outcome (*OR* = 1.38, *p* < .05), suggesting that belonging to the control group was associated with a higher probability of developing mental health problems or a risk for them. Furthermore, age and sex were associated with mental health status. Older compared with younger participants (*OR* = 0.91, *p* = .01) and girls compared with boys (*OR* = 1.40, *p* < .05) showed a higher probability of developing mental health problems or a risk for them. School type and socioeconomic status were not significant.

**Table S2.** Regression Results for Mental Health Status at the 12-Month Follow-Up

|  | *B* | *SE* (*B*) | Wald | *p* | *OR* [95% CI] |
| --- | --- | --- | --- | --- | --- |
| Group^a^ | 0.32 | 0.14 | 5.19 | .023 | 1.38 [1.05, 1.81] |
| Age | -0.10 | 0.04 | 6.58 | .010 | 0.91 [0.84, 0.98] |
| Sex^b^ | 0.34 | 0.15 | 5.08 | .024 | 1.40 [1.05, 1.89] |
| School type^c^ | 0.07 | 0.16 | 0.21 | .649 | 1.07 [0.78, 1.47] |
| Socioeconomic status^d^ | 0.23 | 0.17 | 1.83 | .176 | 1.26 [0.90, 1.76] |
| Constant | 1.08 | 0.73 | 2.20 | .138 | 2.96 [0.70, 12.31] |

*Note*. *N* = 1,116. Mental health status (0 = at risk or with mental health problems, 1 = without mental health problems). ^a^1 = control group, 2 = *StresSOS* intervention group. ^b^1 = female, 2 = male. ^c^1 = Gymnasium (secondary school leading to higher education/university entrance qualification), 2 = others (secondary schools leading to the lower or intermediate school leaving certificate or to the completion of vocational training). ^d^1 = low/medium, 2 = high.

**Group Comparisons as a Function of Program Adherence**

To explore whether there are subgroups that show especially low program adherence, among the *StresSOS* intervention group (*n* = 535; Figure 1), comparisons between participants who utilized at least one *StresSOS* session and one monitoring questionnaire (per-protocol subsample, *n* = 84; indicating higher adherence) and those who did not use at least one *StresSOS* session and one monitoring (intention-to-treat sample without per-protocol subsample, *n* = 451; indicating lower adherence) were performed on sociodemographic variables. Chi-square tests were calculated for categorical data and independent *t*-tests for continuous data (two-sided, α = .05).

Results are presented in Table S3. There was no significant group difference for age (*p* = .16) or school type (*p* = .12). A small difference was observed for sex (*p* = .04, Cramer’s *V* = 0.096) and for socioeconomic status (*p* = .04, Cramer’s *V* = 0.098). Boys and participants with low/medium socioeconomic status were proportionally less represented in the per-protocol subsample which indicates slightly lower adherence among these two groups.

**Table S3.** Comparisons Between Groups (*N* = 535)

|  | *StresSOS* per-protocol use^c^ | | |  |  |
| --- | --- | --- | --- | --- | --- |
| Variable | Yes (*n* = 84) | No (*n* = 451) |  | | |
| Age, *M* (*SD*) | 14.73 (1.90) | 14.42 (1.81) | *t*(533) = 1.40, *p* = .163 | | |
| Sex, *n* (%) |  |  | χ²(1) = 4.36, *p* = .037 | | |
| Female (*n* = 331) | 61 (18.43) | 270 (81.57) |  | | |
| Male (*n* = 204) | 23 (11.27) | 181 (88.73) |  | | |
| Socioeconomic status^a^, *n* (%) |  |  | χ²(1) = 4.44, *p* = .035 | | |
| Low/medium (*n* = 90) | 7 (7.78) | 83 (92.22) |  | | |
| High (*n* = 445) | 77 (17.30) | 368 (82.70) |  | | |
| School type^b^, *n* (%) |  |  | χ²(1) = 2.37, *p* = .124 | | |
| Others (*n* = 155) | 18 (11.61) | 137 (88.39) |  | | |
| Gymnasium (*n* = 379) | 66 (17.41) | 313 (82.59) |  | | |

*Note*. ^a^Socioeconomic status was recorded with the *Family Affluence Scale* (Boyce, Torsheim, Currie, & Zambon, 2006).

^b^No information on the school type was available for one person in the not per-protocol use group. Other school types: secondary schools leading to the lower or intermediate school leaving certificate or to the completion of vocational training. Gymnasium (secondary school leading to higher education/university entrance qualification).

^c^Students who utilized at least one *StresSOS* session and one monitoring questionnaire were assigned to “per-protocol use Yes”. Students who did not meet this criterion were assigned to “per-protocol use No”, indicating less adherence.

**References**

Boyce, W., Torsheim, T., Currie, C., & Zambon, A. (2006). The family affluence scale as a measure of national wealth: Validation of an adolescent self-report measure. *Social Indicators Research, 78*(3), 473–487.

Evensen, M. (2019). Adolescent mental health problems, behaviour penalties, and distributional variation in educational achievement. *European Sociological Review, 35*(4), 474–490.

Kieling, C., Buchweitz, C., Caye, A., Silvani, J., Ameis, S. H., Brunoni, A. R., et al. (2024). Worldwide prevalence and disability from mental disorders across childhood and adolescence: Evidence from the global burden of disease study. *JAMA Psychiatry* 81, 347–356.

Klipker, K., Baumgarten, F., Göbel, K., Lampert, T., & Hölling, H. (2018). Psychische Auffälligkeiten bei Kindern und Jugendlichen in Deutschland – Querschnittergebnisse aus KiGGS Welle 2 und Trends [Mental health problems in children and adolescents in Germany – Cross-sectional results from KiGGS Wave 2 and trends]. *Journal of Health Monitoring, 3*(3), 37–45.

Solmi, M., Radua, J., Olivola, M., Croce, E., Soardo, L., Salazar de Pablo, G., et al. (2022). Age at onset of mental disorders worldwide: Large-scale meta-analysis of 192 epidemiological studies. *Molecular Psychiatry, 27*(1), 281–295.

# Appendix S6

## Fisher's Exact Test Calculated Separately for the Different Measures Used to Create the Primary Outcome Mental Health Status

Individual Fisher’s exact tests (two-sided, α = .05) were calculated for each measure of the combined primary outcome mental health status (using predefined criteria for the assignment to “without problems” vs. “at risk or with problems”). At the 12-month follow-up, in the *StresSOS* intervention group compared with the control group, there were significantly fewer transitions to depressive symptoms as indicated by the PHQ total score (*p* < .05; see Table S4).

**Table S4.** Fisher’s Exact Test for Each Measure of the Combined Primary Outcome Mental Health Status at the 12-Month Follow-Up

| Measure | *OR* [95% CI] | | *p* |
| --- | --- | --- | --- |
| SDQ (total difficulties score) | | 1.13 [0.57, 2.26] | .75 |
| WCS | | 1.38 [0.80, 2.43] | .24 |
| CRAFFT-d | | 1.07 [0.68, 1.68] | .83 |
| AUDIT | | ∞ [0.18 - ∞] | .50 |
| PHQ-A | | 1.58 [1.12, 2.23] | < .05 |

*Note*. SDQ = *Strength and Difficulties Questionnaire* (Goodman & Goodman, 2009). WCS = *Weight Concerns Scale* (Killen et al., 1994). PHQ-A = *Patient Health Questionnaire-9 modified for Adolescents* (Johnson, Harris, Spitzer, & Williams, 2002). CRAFFT-d = *Car, Relax, Alone, Forget, Friends, Trouble questionnaire* (Tossmann, Kasten, Lang, & Strüber, 2009). AUDIT = *Alcohol Use Disorders Identification Test* (Babor, Higgins-Biddle, Saunders, & Monteiro, 2001). The following thresholds were used: SDQ total difficulties score marginal to severe ≥ 20, WCS total score severe symptoms ≥ 58, PHQ-A total score severe symptoms ≥ 10, CRAFFT-d total score severe problems ≥ 2, AUDIT total score severe problems ≥ 20.

References

Babor, T. F., Higgins-Biddle, J. C., Saunders, J. B., & Monteiro, M. G. (2001). *AUDIT: The Alcohol Use Disorders Identification Test: Guidelines for use in primary care* (2nd ed.). Geneva: World Health Organization.

Goodman, A., & Goodman, R. (2009). Strengths and difficulties questionnaire as a dimensional measure of child mental health. *Journal of the American Academy of Child and Adolescent Psychiatry, 48*(4), 400–403.

Johnson, J. G., Harris, E. S., Spitzer, R. L., & Williams, J. B. (2002). The patient health questionnaire for adolescents: Validation of an instrument for the assessment of mental disorders among adolescent primary care patients. *Journal of Adolescent Health, 30*(3), 196–204.

Killen, J. D., Taylor, C. B., Hayward, C., Wilson, D. M., Haydel, K. F., Hammer, L. D., et al. (1994). Pursuit of thinness and onset of eating disorder symptoms in a community sample of adolescent girls: A three-year prospective analysis. *International Journal of Eating Disorders, 16*(3), 227–238.

Tossmann, P., Kasten, L., Lang, P., & Strüber, E. (2009). Bestimmung der konkurrenten Validität des CRAFFT-d: Ein Screeninginstrument für problematischen Alkoholkonsum bei Jugendlichen [Determination of the concurrent validity of the CRAFFT-d: A screening instrument for problematic alcohol consumption in adolescents]. *Zeitschrift für Kinder- und Jugendpsychiatrie und Psychotherapie, 37*(5), 451–459.
