# Supplementary material for: Effectiveness of the school‐based internet intervention StresSOS for the prevention of mental health problems in young people: a randomized controlled trial as part of the ProHEAD consortium
Source: J Child Psychol Psychiatry. 2026 Mar 13;67(8):1393–403. doi: 10.1111/jcpp.70145 (PMC13341385; doi:10.1111/jcpp.70145)
Supplement: Supplementary file 2 — Figure S1. Group assignment for the primary outcome. [file JCPP-67-1393-s002.pptx]

## Slide 1
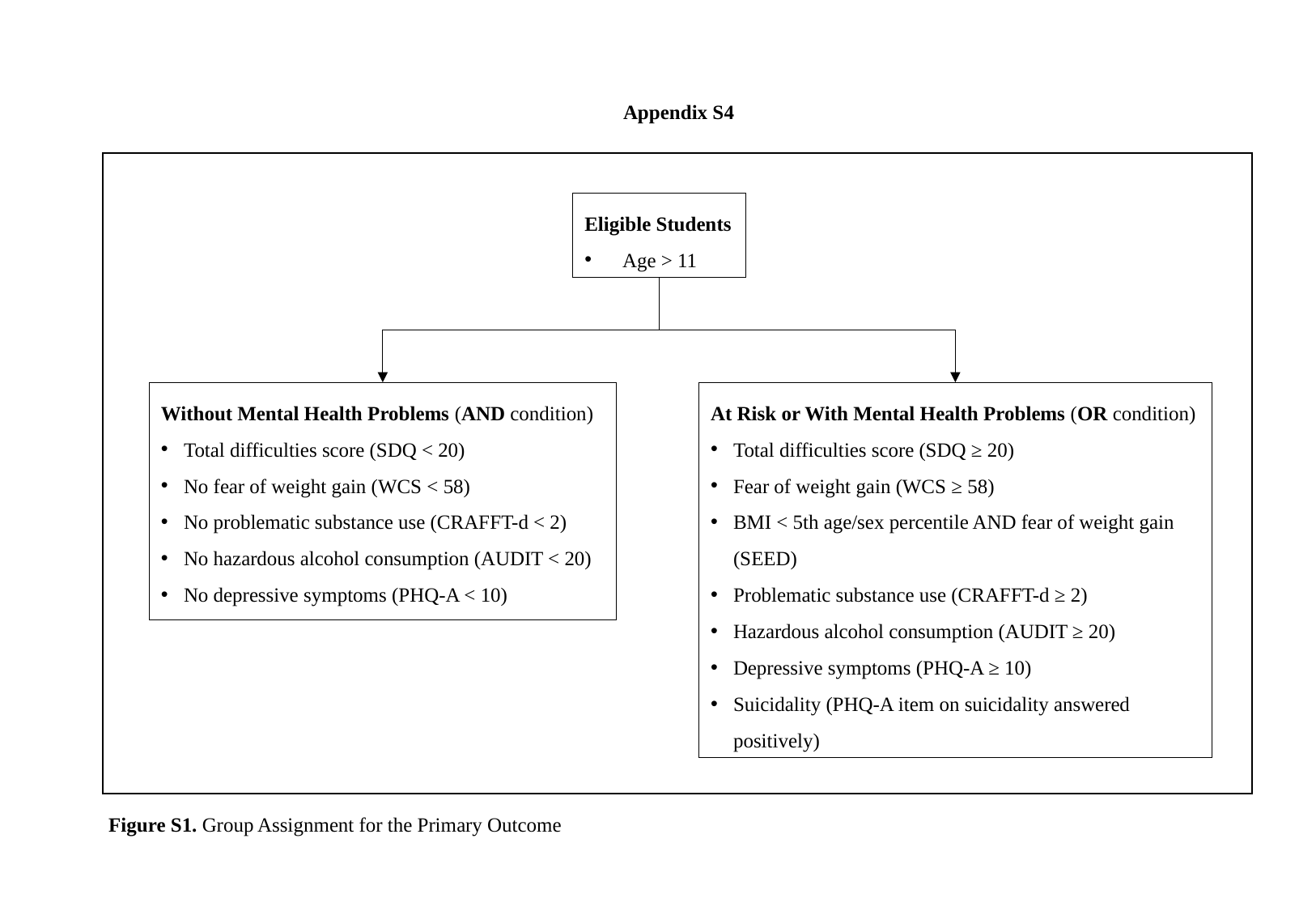

Appendix S4
Eligible Students
Age > 11
Without Mental Health Problems (AND condition)
Total difficulties score (SDQ < 20)
No fear of weight gain (WCS < 58)
No problematic substance use (CRAFFT-d < 2)
No hazardous alcohol consumption (AUDIT < 20)
No depressive symptoms (PHQ-A < 10)
At Risk or With Mental Health Problems (OR condition)
Total difficulties score (SDQ ≥ 20)
Fear of weight gain (WCS ≥ 58)
BMI < 5th age/sex percentile AND fear of weight gain (SEED)
Problematic substance use (CRAFFT-d ≥ 2)
Hazardous alcohol consumption (AUDIT ≥ 20)
Depressive symptoms (PHQ-A ≥ 10)
Suicidality (PHQ-A item on suicidality answered positively)
Figure S1. Group Assignment for the Primary Outcome
